# Synergistic Antimicrobial Metal Oxide-Doped Phosphate Glasses; a Potential Strategy to Reduce Antimicrobial Resistance and Host Cell Toxicity

**DOI:** 10.1021/acsbiomaterials.1c00876

**Published:** 2022-02-24

**Authors:** Farah N. S. Raja, Tony Worthington, Lucas P. L. de Souza, Shirin B. Hanaei, Richard A. Martin

**Affiliations:** † College of Health and Life Sciences and Aston Research Centre for Healthy Ageing, Aston University, Aston Triangle, Birmingham B4 7ET, U.K.; ‡ College of Engineering and Physical Sciences, and Aston Institute of Materials Research. Aston University, Aston Triangle, Birmingham B4 7ET, U.K.

**Keywords:** bioactive glasses, antimicrobial, antimicrobial resistance, synergism

## Abstract

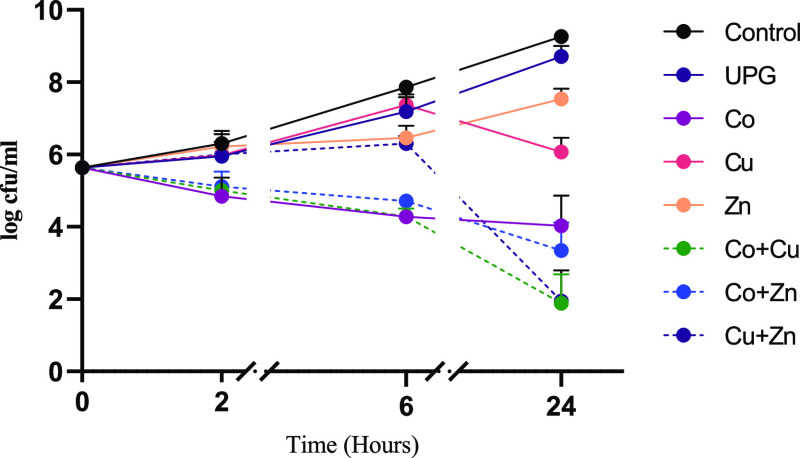

The emergence of antimicrobial resistant strains bacteria and a decline in the discovery of new antibiotics has led to the idea of combining various antimicrobials to treat resistant strains and/or polymicrobial infections. Metal oxide-doped glasses have been extensively investigated for their antimicrobial potential; however to date, most experiments have focused on single metal species in isolation. The present study investigates the antimicrobial potential of sodium calcium phosphates (P_2_O_5_)_50_(Na_2_O)_20_(CaO)_30–*X*_(MO)_*X*_, where M is cobalt, copper, or zinc as single species. In addition, this work studied the effect of co-doping glasses containing two different metal ions (Co + Cu, Co + Zn, and Cu + Zn). The antimicrobial efficacy of all glasses was tested against Gram-positive (*Staphylococcus aureus*) and Gram-negative (*Escherichia coli*) bacterial strains, as well as a fungal strain (*Candida albicans*). Minimum inhibitory and bactericidal concentrations and time kill/synergy assays were used to assess the antimicrobial activity. An enhanced antimicrobial effect, at 5 mg/mL concentration, was exhibited by cobalt, copper, and zinc oxide glasses alone and in combinations. A synergistic antimicrobial effect was observed by Cu + Co and Cu + Zn against *E. coli* and Cu + Zn against *S. aureus*.

## 1 Introduction

Every day millions of patients are prescribed antibiotics to treat infections, which in some cases prove effective; however, inappropriate and overuse of antibiotics has led to a drastic increase in antibiotic resistance. The rapid and continuous emergence of multidrug-resistant strains has shifted the focus away from conventional antibiotics and onto the development of novel antimicrobial agents. Phosphate-based glasses, due to their ability to dissolve at a constant rate and their nontoxic nature, have gained significant interest as localized delivery systems to deliver antimicrobial metal ions directly to the site of interest. In recent years, bioactive glasses doped with metal oxides such as cobalt,^1^ copper^3−5^^,^ gallium,^6,7^ silver,^2,8−10^ and zinc^11^ have been widely investigated for their antimicrobial efficacy against a range of clinically significant microorganisms, both in planktonic form and biofilms.

Antibiotics have been combined to enhance their effects since the 1950s when streptomycin was added with penicillin G to treat enterococcal endocarditis.^12^ Two or more antibiotics can work simultaneously to be additive, synergistic, antagonistic, or have no effect on each other.^13^ The interaction between any two antibiotics is considered synergistic if the combined effect is stronger than an additive expectation and antagonistic if it is weaker.^14^ Several antimicrobials, including two antibiotics, antimicrobial peptides with conventional antibiotics, lactic acid with copper, and copper with Quaternary Ammonium Cations, have been combined and resulted in synergism, demonstrating the beneficial effect of combining antimicrobials in clinical medicine.^15−19^

Combining two antimicrobials not only potentially broadens the spectrum of coverage, as different antimicrobials have their unique cellular targets, which is believed to be more effective than a single target, but it can help fight against multidrug resistant strains.^20^ Treatment of polymicrobial infections with antibiotic combinations has also been documented.^21,22^ Furthermore, combination treatments could also reduce the potential cytotoxic effects of a drug by reducing the concentrations required to inhibit microbial growth for any individual component. As stated earlier, transition metals have been used to dope bioactive glasses for many biomedical applications. However, to date, there has been no study undertaken on the antimicrobial effect of combining two or more metal oxides in a bioactive glass system. It would be advantageous to elucidate whether metal ions have the ability to work synergistically and whether the times required to kill or inhibit microorganisms are reduced. Thus, the aim of this study was to determine if co-doping phosphate-based glass with cobalt, copper, and/or zinc combinations will enhance their antimicrobial effect.

## 2 Experimental Section

### 2.1 Glass Preparation

Glasses were prepared using P_2_O_5_ (99%, Fisher Scientific), NaH_2_PO_4_ (Sigma-Aldrich, Dorset, UK), and CaCO_3_ (99.95%, Alfa Aesar, Lancashire, UK) as starting materials. The undoped glass had a nominal composition of (P_2_O_5_)_50_(Na_2_O)_20_(CaO)_30_. Glasses doped with cobalt, copper, and zinc were prepared using CoO, CuSO_4_ (99%, Sigma-Aldrich, Dorset, UK), and ZnO (99%, Fisher Scientific, UK), respectively. In our previous studies, we characterized sodium calcium phosphate glasses doped with 1, 3, 5, and 10% of CoO^1^ and ZnO.^11^ As expected, an increase in antimicrobial activity was observed with the increasing metal oxide content. A slight cytotoxicity was observed for glasses doped with 10% ZnO. Therefore, sodium calcium phosphate glasses doped with 5 mol % metal oxide (ZnO, CoO and CuO) were selected for this study. Glasses were prepared with 5 mol % metal oxide for each of the three single metal oxides, as shown in Table 1. To evaluate the effect of incorporating two different metal oxides in a single melt quench glass, glasses of the form (P_2_O_5_)_50_(Na_2_O)_20_(CaO)_20_(MO)_5_(M′O)_5_ were prepared where MO and M′O represent CoO, ZnO, or CuO. The combinations investigated were Co + Zn, Co + Cu, and Cu + Zn, as shown in the Table 1.

**Table 1:** Glass Compositions for Evaluating the Synergistic Potential

mol %						
	P_2_O_5_	CaO	Na_2_O	CoO	CuO	ZnO
undoped (UPG)	50	30	20	0	0	0
Co	50	25	20	5	0	0
Cu	50	25	20	0	5	0
Zn	50	25	20	0	0	5
Co + Cu	50	20	20	5	5	0
Co + Zn	50	20	20	5	0	5
Cu + Zn	50	20	20	0	5	5

Precursors were weighed out, mixed thoroughly, and placed into a 59 mL 90% Pt–10% Rh crucible (GLC alloys Ltd Middlesex, UK). The crucible and reagents were then placed in the furnace at room temperature and heated to 300 °C, at a ramp rate of 10 °C per minute, and after reaching the desired temperature, the reagents were allowed to dwell for 1 h. The temperature was then increased to 600 °C, at a ramp rate 60 °C per minute, and the reagents were left to dwell for 30 min. Finally, the temperature was rapidly increased (60 °C per minute) up to its maximum melting temperature of 1050 °C, and the sample was held at this temperature for 30 min. The molten liquid was then poured into a preheated (350 °C) graphite mold and annealed overnight before being slowly cooled to room temperature. The resultant glasses were ground using a mortar and pestle and sieved to produce particle size ranging from 40–60 μm. Prior to undertaking the experiments, glass powders were sterilized using a dry heat at 180 °C for 2 h. Samples were stored in a desiccator between stages of preparation to reduce exposure to atmospheric moisture.

### 2.2 Physiochemical Characterization

#### 2.2.1 X-ray Diffraction Analysis

X-ray diffraction was carried out to determine the amorphous nature of the manufactured glasses. The experiments were carried out at the I-15 beamline at the Diamond Light Source, Harwell, UK. The instrument was set up to collect data in a 2θ geometry with a Si monochromatic, and the energy of the X-ray beam was 76.7 keV, λ = 0.162 Å. Finely ground glass powders were loaded in to 1.17 mm × 1.5 × 40 mm SiO_2_ glass capillaries at room temperature and mounted on the sample changer (placed at right angles to incident X-ray beam). An empty capillary was measured to account for background corrections. Data corrections and normalizations were carried out using GUDRUNX.

#### 2.2.2 Energy-Dispersive X-ray Spectroscopy

Spectra were collected for each of the samples using a JCM-6000PLUS (JEOL) with a JED-2300 Analysis Station operating at 15 keV. Samples were coated with carbon to ensure conductivity. The ZAF correction method was used to provide quantitative analysis.

#### 2.2.3 Ion Release Study

The cation concentration (Co^+2^, Cu^+2^, and Zn^+2^) following 24 h incubation was studied using inductively coupled plasma optical emission spectrometry (iCAPTM 7000 Plus Series). Five mol % glasses samples at 5 mg/mL concentration were incubated with distilled water for 24 h at 37 °C. The dissolution products were then filtered using 0.2 μm Ministart filters (Fisher Scientific, UK), and samples were run. The concentration of each ion was calculated from the linear portion of the generated standard curve as ppm.

### 2.3 Microbial Strains

Two bacterial strains, *Escherichia coli* (NCTC 10538) and *Staphylococcus aureus* (ATCC 6538), and a fungal strain, *Candida albicans* (ATCC 76615), were used in this study. These strains were maintained at −80 °C on MicroBank beads (Pro-Lab Diagnostics Neston, Cheshire, UK). *E. coli* and *S. aureus* were cultured in nutrient broth/agar and incubated at 37 °C, whereas *C. albicans* was maintained in Sabouraud dextrose broth/agar (SDB/SDA) at 30 °C. Initial cultures were prepared by inoculation of respective broth with a single colony of test strain. Following inoculation, the broth was incubated for 24 h under aerobic conditions.

### 2.4 Determination of Minimum Inhibitory and Bactericidal Concentrations

Broth macrodilution was performed in accordance with the Clinical & Laboratory Standards Institute (CLSI) guidelines to determine minimum inhibitory concentration (MIC) and minimum bactericidal concentration (MBC) of the glass particles.^23^ Double dilutions of each stock solution (100 mg/mL in sterile phosphate-buffered saline) were performed in the range from 50–0.2 mg/mL by adding 1 mL of Mueller Hinton Broth (Oxoid Ltd, U.K.). To maintain equal volumes throughout the procedure, 1 mL of solution was discarded from the last dilution. Triplicate samples were prepared in sterile Bijoux bottles (Thermo Scientific, U.K.), inoculated with 10^5^ cfu/mL, and incubated overnight in a shaking incubator at 200 rpm at 37 °C; *C. albicans* at 30 °C in an aerobic environment. Under the same growth condition, solution devoid of glass dissolution products was used as a growth control. To avoid potential misinterpretation of turbidity due to the colored solution of glasses, liquid medium without microorganisms but containing the same concentration of glass dissolution products was used as standard solutions for comparison.

After overnight incubation, the turbidity of the test solutions was checked against glass standard solutions. MIC was determined as the lowest concentration, which showed no turbidity on visual inspection. One hundred μL of the lowest concentration that was not visually turbid i.e., MIC and higher concentrations than MIC along with the controls were plated onto Mueller Hinton agar and incubated overnight at 37 °C; *C. albicans* at 30 °C on SDA in an aerobic environment. MBC was determined as the lowest concentration, which yielded three log reductions i.e., a 99.9% reduction in cfu/mL compared to the control. Tests were performed in triplicates and repeated three times.

### 2.5 Evaluation of Synergistic Antimicrobial Effect Using Time Kill Assay

Time kill assays were used to determine the antimicrobial effect of glasses over a time period. The aim of the assay was to test metal oxides alone and in combination to elucidate their synergistic antimicrobial potential. This was assessed using a suspension method, as described by White and co-workers.^24^ Co-doped glasses containing two different metal ions (Co + Cu, Co + Zn, and Cu + Zn) were studied for their antimicrobial potential.

Each strain was tested against 5 mol % cobalt-, copper-, and zinc-doped glasses, alone and in combination at 5 mg/mL. Experimental suspensions for each sample were seeded with an initial microbial density of 10^5^ cfu/mL. A control containing Mueller–Hinton broth seeded with a microbial inoculum was included as a growth control for each isolate, whereas a negative control (broth without glass or test strain) was also included. All tubes were incubated aerobically at 37 °C or 30 °C for 24 h in a shaking incubator at 200 rpm. At time periods 0, 2, 6, and 24 h, 100 μL aliquots were diluted 1:10 in D/E neutralization buffer to prevent antimicrobial carryover. Diluted samples were subcultured on Mueller Hinton agar and incubated overnight at 37 °C or 30 °C under aerobic conditions after which cfu were determined. Synergy was defined as a ≥2 log_10_ reduction in the colony count between the combination and the most active agent at 24 h. Additive or indifference was a <2 log_10_ decrease in the colony count at 24 h by the combination compared with the most active single agent, whereas antagonism was a ≥2 log_10_ increase in the colony count after 24 h between the combination and the most active agent.^37^

### 2.6 Cytotoxicity Assay

Human Osteosarcoma cells (SAOS-2, ATCC HTB-85) were cultured in McCoy’s 5A Medium (ATCC 30–2007) supplemented with 15% fetal bovine serum (FBS) (ATCC 30–2020). Cells were kept in a cell incubator at 37 °C in an atmosphere of 5% CO_2_. Keratinocytes (HaCaT, Caltag Medsystems Ltd) were cultured in Dulbecco’s Modified Eagle Medium (DMEM) Medium—High Glucose (Gibco) supplemented with 10% FBS. To test the cytocompatibility of the proposed biomaterials, culture media were conditioned with 5 mg/mL of each type of glass. An appropriate amount of powder was added to basal media, mixed for 24 h, and filtered using an ultrafine filter (0.22 μm pore size). Only after filtration, the appropriate volume of FBS was added to the glass-conditioned media, which were left in the cell incubator overnight to acclimatize and buffer their pH before being used to treat cells.

For the cytotoxicity experiment, 10,000 cells/cm^2^ were seeded in 96-well plates. Cells were then treated with the glass-conditioned media for 24 h. Cells incubated in their appropriate growth medium and cells killed by 30-min incubation in 70% ethanol were used as controls. Following the experimental time, a methylthiazolyldiphenyl tetrazolium bromide (MTT) assay was performed. Briefly, all media were removed from every well and replaced with 100 μL of a 1:10 (1.2 mM) solution of MTT and phenol-free DMEM (GibcoTM), and the plates were incubated for 4 h at 37 °C. The precipitated formazan was dissolved by replacing 75 μL from each well with 50 μL of dimethyl sulfoxide (Invitrogen) and incubating for 10 min. Optical density was measured at 540 nm using a microplate reader (Thermo, Multiskan GO). This experiment was performed in triplicate for each time point.

### 2.7 Statistical Analysis

Two-way analysis of variance was carried out to determine statistical significances (GraphPad Prism 8.4.2). If a significant difference was detected, a Tukey test was carried out to determine which values were significantly different. Differences were considered statistically significantly at a level of *P* < 0.05.

## 3 Results

### 3.1 Glass Manufacturing

**Figure 1:**
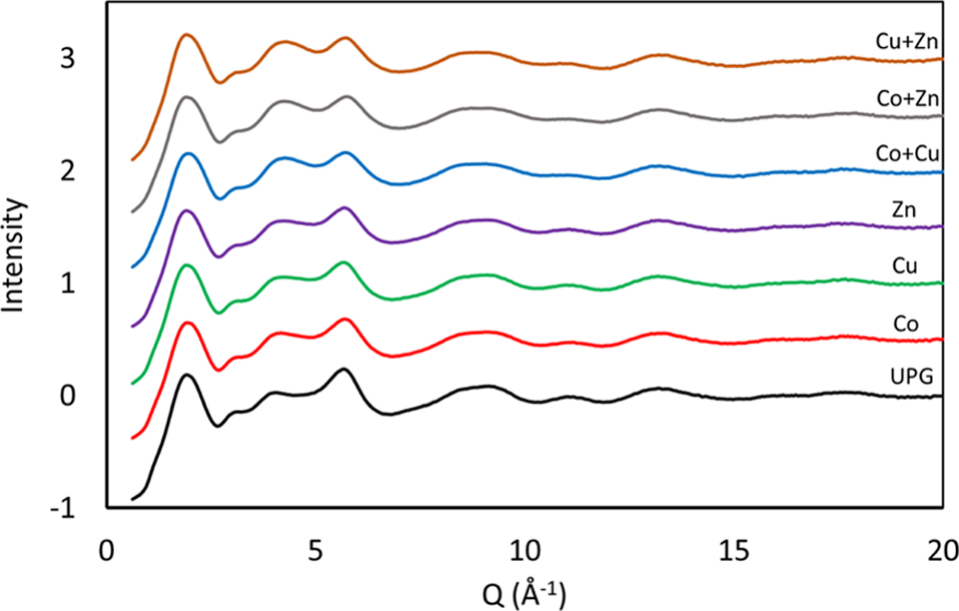
X-ray diffraction spectra, illustrating the absence of Bragg peaks.

A series of cobalt, copper, and zinc oxide-doped glasses were successfully prepared. During the melt process, calcium carbonate decomposes and releases CO_2_ to give CaO, sodium dihydrogen orthophosphate releases water to give P_2_O_5_ and Na_2_O, while sulfur trioxide was released from copper sulfate to yield copper II oxide (CuO), as reported previously.^25^ Nominal compositions of the resulting oxide glasses are given in Table 1. Batch weights were consistent, with the initial batch size accounting for the release of carbonates, water, and sulfates. The glasses were optically transparent with their expected characteristic colors; clear (zinc and UPG), green (copper), and traditional cobalt blue (cobalt). All the glasses were fully amorphous with no visible signs of Bragg peaks, as shown in Figure 1. Energy-dispersive X-ray spectroscopy (EDS) results were fully consistent with the nominal compositions given in Table 1. Na_2_O values were 19.4 ± 1.8 compared to an expected value of 20.0; CaO values were 25.6 ± 1.1 for samples doped with a single antimicrobial ion compared to an expected value of 25.0, while for samples co-doped with two antimicrobials, CaO values were 19.8 ± 0.8. Phosphate values were all within two standard deviations of 50% as expected.

### 3.2 Ion Release Study

The ion release profile for each glass composition was studied. Synergy is defined as a ≥2 log_10_ reduction in the colony count between the combination and the most active agent at 24 h, and ion release at 24 h is shown in Table 2. No significant difference in the release of cobalt, copper, and zinc was seen for co-doped compositions when compared with glasses doped alone.

**Table 2:** Accumulative Ion Release (ppm) of Cobalt, Copper, and Zinc Following 24 h Incubation in Distilled Water

	Co	Cu	Zn	Na	Ca
undoped (UPG)				465	491
Co	166			483	425
Cu		133		437	400
Zn			187	418	379
Co + Cu	150	140		434	317
Co + Zn	148		149	430	321
Cu + Zn		135	180	419	320

### 3.3 Minimum Inhibitory and Bactericidal Concentrations of Cobalt, Zinc, and Copper Oxide-Doped Glasses Using Broth Microdilution

The Co-, Cu-, and Zn-doped glasses demonstrated a greater antimicrobial activity in comparison to undoped phosphate-based glass. The MIC values of cobalt-doped phosphate glass were considerably lower compared to copper- and zinc-doped phosphate glasses (≤0.78 mg/mL for cobalt-doped glass, 3.13–12.5 mg/mL for copper-doped glass, and 1.5–3.13 mg/mL for zinc-doped glass Table 3). *C. albicans* demonstrated reduced susceptibility to copper compared to the other microorganisms tested, where the highest concentration tested of copper inhibited the growth.

**Table 3:** Antimicrobial Activity (MIC and MBC) of 5 mol % Metal Oxide (Cobalt, Zinc, and Copper)-Doped Phosphate Glasses against *E. coli*, *S. aureus,* and *C. albicans*^a^

	MIC in mg/mL				MBC in mg/mL		
microbial strains	UPG	Co	Cu	Zn	Co	Cu	Zn
*E. coli*	>50	0.39	3.13	1.5	0.78	6.25	3.13
*S. aureus*	>50	0.78	6.25	3.13	3.13	12.50	6.25
*C. albicans*	>50	0.78	12.50	3.13	1.56	25	6.25

a Broth microdilution assay was performed in the range of 50–0.2 mg/mL. (MIC: minimum inhibitory concentration and MBC: minimum bactericidal concentration).

### 3.4 Evaluation of the Antimicrobial Activity of Co-doped Phosphate-Based Glasses

**Figure 2:**
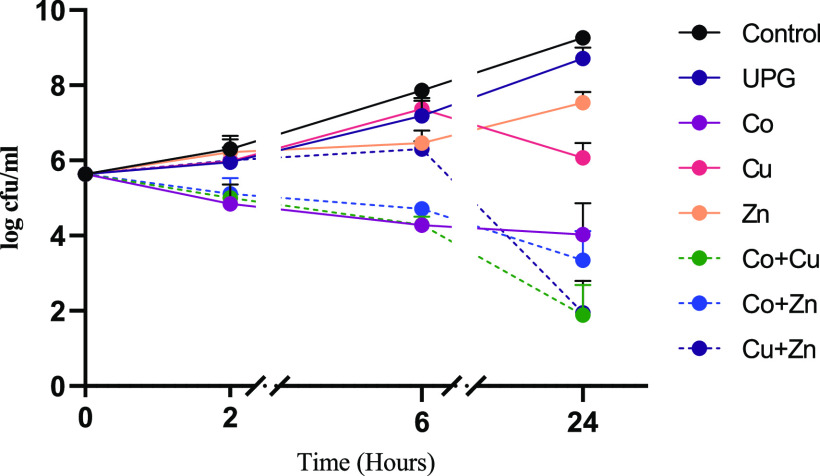
Time kill curves of undoped phosphate glass (UPG) and 5 mol % cobalt, zinc, and copper oxide-doped phosphate glass powders at 5 mg/mL against *E. coli*. The combinations investigated were Co + Cu, Co + Zn, and Cu + Zn. Microbial viability is presented as log cfu/mL. Data shown are expressed as mean ± SD (*N* = 3).

Time kill curves of cobalt-, copper-, and zinc oxide-doped glasses alone and combined (50/50 mol %) against *E. coli* are shown in Figure 2. A significant antimicrobial effect was seen for cobalt-doped glasses in as little as 2 h (*p* = 0.0043), whereas the significant effect by zinc-doped glass was seen at 6 h (*p* = 0.0088). Copper-doped glass failed to demonstrate a significant decrease at 2 and 6 h, and significance was only seen at 24 h (*p* < 0.001). When considering the effect of combining two different glasses (5 mol % of each dopant), a synergistic activity was seen for Co + Cu and Cu + Zn, with a greater than 2 log reduction in colony count being observed at 24 h compared to respective individual metal oxide-doped glasses alone.

**Figure 3:**
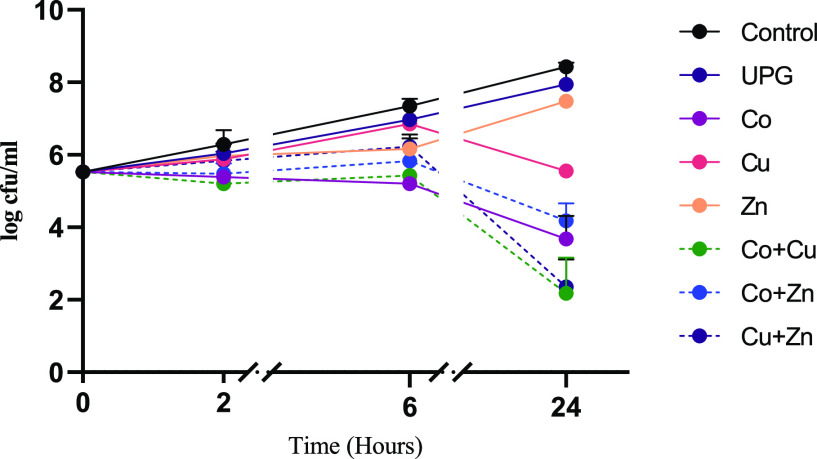
Time kill curves of UPG and 5 mol % cobalt-, zinc-, and copper oxide-doped phosphate glass powders (5 mg/mL), in comparison to the untreated control, alone, and in combination against *S. aureus*. The combinations investigated were Co + Cu, Co + Zn, and Cu + Zn. Microbial viability is presented as log cfu/mL. Data shown are expressed as mean ± SD (*N* = 3).

Figure 3 shows the antimicrobial effect of combining two metal oxides in a single phosphate glass system against *S. aureus*. A significant decrease in the bacterial count was seen at 2 h for 5 mol % cobalt-doped glass (*p* = 0.0376), whereas a non-significant difference was observed for 5 mol % copper- and zinc-doped glasses at this early time point. A significant decrease in bacterial count was shown by all glasses at 24 h. The combined effect of glasses showed that Cu + Zn has a synergistic effect against *S. aureus*, whereas Co + Cu and Co + Zn showed indifference.

**Figure 4:**
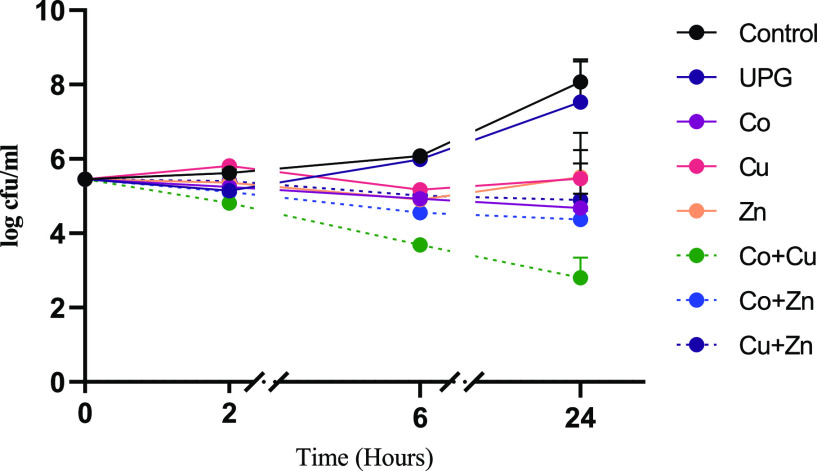
Time kill curves of UPG and 5 mol % cobalt-, zinc-, and copper oxide-doped phosphate glass powders (5 mg/mL), in comparison to the untreated control, alone, and in combination against *C. albicans*. The combinations investigated were Co + Cu, Co + Zn, and Cu + Zn. Microbial viability is presented as log cfu/mL. Data shown are expressed as mean ± SD (*N* = 3).

Figure 4 shows the time kill curves of cobalt-, copper-, or zinc-doped phosphate-based glasses, alone and in combination against *C. albicans*. A significant decrease in the growth of *C. albicans* was seen at 24 h when treated with cobalt-, copper-, or zinc oxide-doped glasses alone (*p* < 0.0001). However, the antimicrobial effect of glasses was less pronounced against *C. albicans* than *E. coli* or *S. aureus* (Figures 2 and 3). Combining metal oxides within the glass system, however, showed no synergistic activity against *C. albicans*.

### 3.5 Cytotoxic Evaluation against Human Cells

**Figure 5:**
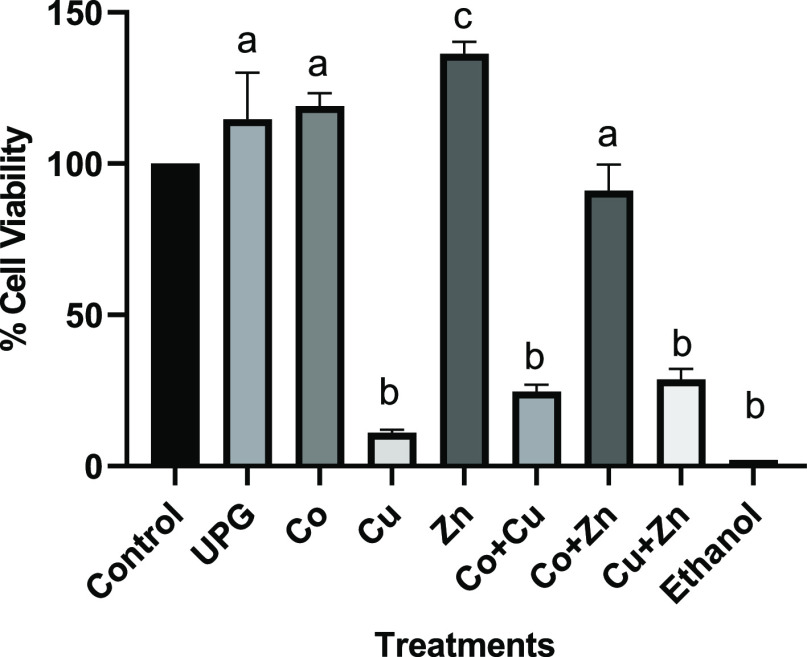
Cell viability of human keratinocytes—HaCaT cells following 24 h treatment with 5 mol % glass compositions alone (Co, Cu, and Zn) and in combination (Co + Cu, Co + Zn, and Cu + Zn). Statistically significant differences between control cells and treatments are indicated by letters a (nonsignificant), b (significant decrease), and c (significant increase).

**Figure 6:**
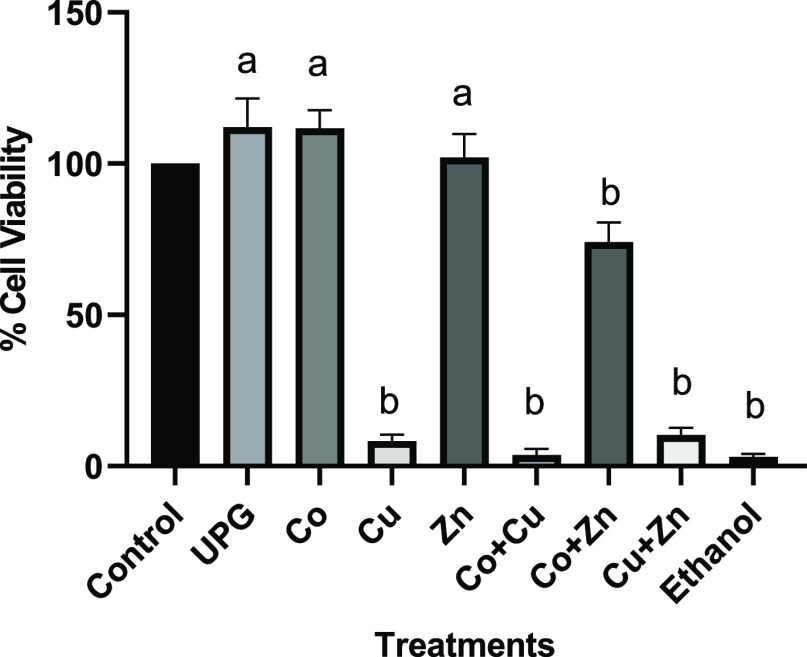
Cell viability of human osteoblast-like cells—Saos-2 following 24 h treatment with 5 mol % glass compositions alone (Co, Cu, and Zn) and in combination (Co + Cu, Co + Zn, and Cu + Zn). Statistically significant differences between control cells and treatments are indicated by letters a (nonsignificant) and b (significant decrease).

The cytotoxicity of the conditioned media containing 5 mol % of cobalt-, copper-, zinc-, and co-doped glasses (Co + Cu, Co + Zn, and Cu + Zn) was determined using MTT assay on human keratinocytes and osteoblast like cells. The cell viability of undoped glass and treatment groups was determined relative to the control cells cultured in respective medium. No cytotoxic effect was seen for the undoped phosphate-based glass composition (UPG), 5 mol % cobalt- and zinc-doped glasses against both cell lines (*p* < 0.05). Five mol % zinc showed a significant increase in HaCaT’s viability, indicative of the possible proliferation effect (*p* = 0.001) of zinc ions, whereas copper-doped glass caused a significant decrease in cell viability (p < 0.0001). Glass combinations Co + Cu and Cu + Zn showed a significant decrease in the cell viability against both cell lines, as shown in Figure 5 and 6 (*p* < 0.0001). Overall, the cytotoxic effect was less pronounced against keratinocytes than osteoblast-like cells.

## 4 Discussion

The polymicrobial nature of various infections and the development of resistant strains have become a substantial clinical problem over the past few decades. Therefore, researchers are striving to gain comprehensive knowledge about drug-resistant strains and assess alternative treatments such as combination prospects. This study adds to the current knowledge of the antimicrobial potential of bioactive glasses and provides an insight into the effect of combining metal oxides within a glass system. The purpose of this study was first to identify which metal oxides are most effective against microorganisms such as *E. coli*, *S. aureus,* and/or *C. albicans* and second to identify combinations of glass compositions with synergistic antimicrobial effects.

Five mol % cobalt-, copper-, or zinc-doped phosphate glasses were tested alone and in combination against clinically relevant microorganisms. The synergistic effect was studied using time kill assays. The results of this study demonstrated that 5 mol % cobalt-, copper-, or zinc oxide-doped phosphate glass compositions, at the concentration of 5 mg/mL, show a strong antibacterial and moderate antifungal activity in the planktonic form. The lack of antimicrobial activity seen for undoped phosphate-based glass demonstrates that the doped glass’s antimicrobial efficacy is derived from the cobalt, copper, or zinc ions. Additionally, this study is the first to report a synergistic antimicrobial effect of metal oxide-doped bioactive glasses. While no synergistic effect was seen against *C. albicans*, Cu + Co and Cu + Zn showed the synergistic effect against *E. coli* and Cu + Zn against *S. aureus*.

The antimicrobial action appeared organism-specific, with *E. coli* being the most sensitive strain, whereas *C. albicans* was least susceptible. This could be attributed to the outer cell wall structure of the microorganisms tested. Gram-negative bacteria, unlike Gram-positive bacteria, have ion channels that allow for penetration of metallic ions through the outer membrane, which subsequently bring about cell death. In addition to thick peptidoglycan, cationic sequestering due to anionic metal binding sites occur on the surface of Gram-positive bacteria, which contributes to resistance of *S. aureus* killing.^26−29^ On the other hand, pathogenic fungi such as *C. albicans* have developed complex mechanisms to regulate the surplus metal ions by expressing importers that sequester excess metal to unique proteins like metallothioneins.^30^ Furthermore, glass dissolution is a complex process, which depends on several factors such as glass composition, structure, dissolving media, pH, and temperature, while the data (Table 2) show that the incorporation of an additional metal oxide in the glass system did not significantly affect the dissolution and ion release profile in distilled water; nevertheless, microbiological media provides a complex environment with a number of ionic species that may reduce the concentration of free metal ions by precipitation or by the formation of soluble complexes.^31^ Nutrient broth and Sabouraud dextrose broth vary in their composition with considerably different concentrations of salts and sucrose, which is likely to affect glass dissolution behavior and hence the biological response.

While some combinations showed “indifference,” that is, lack of synergy, nevertheless, the antimicrobial effect was enhanced when compared to the metals alone. For example, Co + Cu against *C. albicans* did not have a synergistic effect, but the antimicrobial effect of co-doped glass was greater (5.2 log reduction) than the cobalt (3.4 log reduction)- and copper oxide (2.6 log reduction)-doped glasses alone. In addition, co-doped glasses started to show significant antimicrobial activity as early as 2 h. These findings demonstrate the beneficial effects of co-doping metal oxides within a glass system. Bioactive glasses are generally believed to have a broad-spectrum antimicrobial action; however, various metal ions have also been shown to target specific cell components or processes. For instance, copper ions are believed to target the cell membrane,^32^ whereas cell death due to cobalt ions is brought about due to hypoxic conditions.^33,34^ Therefore, co-doping with metal oxides is also likely to broaden the antimicrobial coverage of bioactive glasses as well as reduce the likelihood of microorganisms developing resistance.

One primary concern of antibiotics is drug toxicity; therefore, the cytotoxic effect of the single and co-doped glass dissolution products was assessed against human dermal keratinocytes (HaCaT) and osteoblast-like cells (Saos-2 cells). Previous studies have shown adverse effects of higher cobalt concentrations on mammalian cells.^35−37^ However, our data showed a nonsignificant difference in the viability of both keratinocytes and osteoblasts when exposed to cobalt-doped glasses, and a proliferative effect of zinc-doped glasses was observed. On the contrary, copper-doped glass alone and co-doped compositions demonstrated significant toxicity. Copper has been shown as one of the most promising dopants not only for its antimicrobial properties but also for bone regeneration and angiogenic potential.^38^ It is fundamental to human health, and living organisms have evolved complex mechanisms to eliminate the excess; therefore, it is considered safe by many authors.^39^ Previous studies have demonstrated a significant antimicrobial effect of copper-doped glasses at 5 mol %, 8.5 wt %, and 10 mol %; however, these studies lack cytotoxicity data^25,40,41^ and are therefore difficult to assess the potential of those materials. Conversely, lowering the mol % of metal oxides could possibly reduce cytotoxicity, while maintaining the antimicrobial effect. Previously, 2 mol % Cu-doped glass scaffolds have been shown to induce osteogenesis, while inhibiting infections.^42^ Our study highlights the significance of cytotoxicity studies, as it can vary depending upon the doses and target tissue. As the release of ionic species from the bioactive glasses varies with the mol % of the constituent metal oxides, which determines the biological response, these glasses could be modified by lowering the dopant’s concentration to achieve therapeutic benefits.

## 5 Conclusions

Five mol % cobalt- and copper oxide-doped phosphate glass (5 mg/mL) demonstrated a strong antimicrobial activity against Gram-positive and -negative bacteria, as well as *C. albicans*. Zinc oxide-doped glasses showed a moderate response against all strains. A synergistic antimicrobial efficacy was shown by Cu + Co and Cu + Zn against *E. coli* and Cu + Zn against *S. aureus*. Copper and copper co-doped compositions containing 5 mol % copper oxide showed cytotoxic effects against human cells, suggesting that lower concentrations are required. Nevertheless, cobalt- and zinc oxide-doped glasses at 5 mol % show promising antimicrobial results with minimum or no cytotoxicity.

## Notes

DATA AVAILABLITY STATEMENT: The supporting data of the study are available from the corresponding author upon request.
